# Impact of mandatory online learning module for healthcare workers intending to decline influenza immunization: Implications for coronavirus disease 2019 (COVID-19)

**DOI:** 10.1017/ash.2021.174

**Published:** 2021-07-28

**Authors:** Leanne M. Delaney, Victoria R. Williams, Nick Tomiczek, Lawrence Robinson, Alex Kiss, Jerome A. Leis

**Affiliations:** 1 Department of Medicine, University of Toronto, Toronto, Ontario, Canada; 2 Sunnybrook Health Sciences Centre, Toronto, Ontario, Canada; 3 Sunnybrook Research Institute, Sunnybrook Health Sciences Centre, Toronto, Ontario, Canada; 4 Centre for Quality Improvement and Patient Safety, University of Toronto, Toronto, Ontario, Canada

## Abstract

A policy mandating the completion of an online learning module for healthcare workers intending to decline influenza immunization was associated with a nearly 25% relative increase in immunization and significant reduction in healthcare-associated influenza. In the absence of mandatory vaccination, this model may help to augment severe acute respiratory coronavirus virus 2 (SARS-CoV-2) vaccine efforts.

Healthcare worker (HCW) immunization is the most important intervention for reducing healthcare-associated influenza (HAI) and associated mortality.^1^ Early evidence suggests that severe acute respiratory coronavirus virus 2 (SARS-CoV-2) immunization strongly reduces the incidence of healthcare-associated coronavirus disease 2019 (COVID-19).^2^ Despite these benefits, vaccine hesitancy among HCWs frequently leads to suboptimal institutional immunization rates.^3,4^ Strategies promoting awareness, optional education, and accessibility of vaccines fall short of achieving immunization rates required to significantly reduce HAIs.^5^ Mandatory HCW immunization achieves immunization rates >90%, but acceptance of this strategy is challenging.^6–8^ A softer policy includes mandating a decision on immunization by requiring HCWs to sign a declination statement to refuse immunization.^9^ This strategy is more feasible, but significant variability exists in how it is implemented, with simple declination forms resulting in modest increases in vaccine uptake.^7,10^

The availability of SARS-CoV-2 vaccine highlights an urgent need to define how best to implement mandatory decision policies to optimize HCW immunization rates. We hypothesized that a declination statement can nudge HCWs more effectively toward immunization when linked to a mandatory online learning module. We implemented a corporate influenza vaccine policy prior to the COVID-19 pandemic that requires completion of a mandatory online learning module by HCWs intending to decline immunization.

## Methods

A 3-year quasi-experimental study was performed at our multifacility academic health center assessing the impact of this new corporate influenza vaccine policy on HCW vaccine uptake and HAI. At baseline, promotional strategies alone resulted in stable HCW influenza immunization rates between 50% and 60%.

The new policy implemented in 2019–2020 required all hospital staff, with exception of those on leave, to complete one of the following options annually by December 15: (1) receive influenza vaccine; (2) submit a medical certificate from a qualified specialist confirming that the vaccine is medically contraindicated; or (3) sign a declination statement after completing a mandatory online learning module. It was communicated that failure to follow this policy may result in discipline up to and including termination of employment. Managers received compliance reports to follow-up with nonadherent staff. The 15-minute learning module contained a patient story, information about vaccine efficacy and safety, and addressed common myths associated with influenza vaccine hesitancy. Once completed, staff could proceed with vaccination or sign the declination statement. No other changes were made to the influenza vaccination campaign.

All active hospital employees and physicians were included in the vaccine denominator, with the exception of residents, medical students, and volunteers because their data is not stored centrally. All included HCWs were grouped based on facility (acute care, rehabilitation, orthopedic hospital, long-term care, undifferentiated), interaction with patients (patient facing, non–patient facing), location (administrative or support, ambulatory, inpatient), department (medicine, surgery, critical care, psychiatry, obstetrics, outpatient, administration, other), and professional role (nursing, allied health, support, and other clinical which included physicians).

The primary outcome was HCW immunization by December 15 during the baseline season compared to 2 subsequent intervention seasons. The secondary outcome was the rate of HAI per 1,000 patient days, determined based on midturbinate swabs of patients who had new or worsening onset of 1 or more respiratory symptom (rhinorrhea, cough, sore throat, wheeze or dyspnea) >72 hours after admission. HAI analysis was excluded in the 2020–2021 season to minimize confounding bias of the COVID-19 pandemic.

The primary outcome was analyzed using a Poisson regression model that adjusted for season, facility, professional role, location, and level of patient interaction. The secondary outcome was analyzed by comparing aggregate HAI per 1,000 patient days as an incident rate ratio. All analyses were carried out using SAS version 9.4 software (SAS Institute, Cary, NC). This study met criteria for exemption of research ethics review based on our institutional process for confirming that the project was improvement in quality and not human subject research.

## Results

Following implementation of the corporate policy, institutional influenza immunization rates increased from 58% (5,144 of 8,822) in 2018–2019 to 74% (6,267 of 8,494) in 2019–2020 and were sustained at 72% (6,154 of 8,591) in 2020–2021. Table 1 provides the breakdown of immunization rates and HAI by facility and departments. HAI declined during the first year from 0.2 per 1,000 patient days to 0.08 per 1,000 patient days (*P* = .01).

**Table 1.** Healthcare Worker (HCW) Immunization Rates and Healthcare-Associated Influenza (HAI) by Facility and Department Before and After Implementation of Corporate Policy Requiring That All HCWs Make a Decision About Influenza Immunization

Outcomes	Baseline (2018–2019)		Intervention year 1 (2019–2020)		Intervention year 2 (2020–2021)	
	HCW Immunization Rate, No. (%)	HAIs per 1,000 Patient Days	HCW Immunization Rate, No. (%)	HAIs per 1000 Patient Days	HCW Immunization Rate, No. (%)	HAI per 1,000 Patient Days
**Acute care**	2,584 (56.2)	8 (0.15)	3240 (72)	6 (0.12)	3,251 (69.9)	NA
Medicine	359 (57.4)	4 (0.22)	458 (74.8)	5 (0.29)	472 (72.3)	
Surgery	282 (60.3)	2 (0.13)	347 (72.3)	1 (0.07)	339 (72.3)	
Critical care	214 (53.5)	1 (0.11)	233 (58.4)	0	261 (62.7)	
Psychiatry	47 (52.8)	0	74 (77.1)	0	80 (69.6)	
Obstetrics	255 (56.9)	1 (0.13)	335 (73.3)	0	318 (68.1)	
Outpatient	548 (57.6)	NA	720 (75.1)	NA	691 (70.9)	
Administration	452 (48.4)	NA	578 (65.7)	NA	562 (63.4)	
Other	427 (62.8)	NA	495 (80.5)	NA	528 (79)	
**Long-term care**	648 (78.4)	9 (0.19)	670 (86.1)	1 (0.02)	593 (78.9)	NA
Inpatient units	549 (82.7)		563 (88.1)		498 (80.8)	
Other	99 (60.7)		107 (77)		95 (69.9)	
**Rehabilitation**	236 (55.7)	8 (0.47)	287 (71.8)	3 (0.17)	260 (63)	NA
Inpatient units	171 (58.2)		193 (70.2)		174 (62.8)	
Other	65 (50)		94 (75.2)		86 (63.2)	
**Orthopedic hospital**	189 (49.3)	0 (0)	245 (68.4)	0 (0)	259 (66.6)	NA
Inpatient units	119 (48.4)		145 (65.9)		141 (60.8)	
Other	70 (51.1)		100 (72.5)		118 (75.2)	
**Undedicated**	1,487 (57.3)	NA	1,825 (74.2)	NA	1,791 (75)	NA
Physicians	516 (96.1)		502 (91.6)		494 (88.7)	
Corporate	971 (47.2)		1,323 (69.2)		1,297 (70.8)	
**Overall**	5,144 (58.3)	25 (0.2)	6,267 (73.8)	10 (0.08)	6,154 (71.6)	NA

Note. NA, nonapplicable because not inpatient area, or excluded due to confounding of COVID-19 pandemic on HAI.

In both intervention seasons combined, policy adherence was 14,189 (83%), with 12,421 (72.3%) choosing to be immunized without completing the educational module, 19 (0.1%) providing medical exemption, and 1,810 (10.6%) completing the educational module. Following module completion, 61 (3.4%) subsequently received immunization, and 1,749 (96.6%) signed the declination form.

Table 2 summarizes immunization rates by season, professional role, location, level of patient interaction, and facility. Every category improved following the intervention. After adjusting for facility, location, level of patient interaction, and professional role, the policy resulted in a higher HCW immunization rate during the first (IRR, 1.26; 95% CI, 1.21–1.31) and second (IRR, 1.22; 95% CI, 1.18–1.27) years following implementation, without a difference between intervention seasons (IRR, 1.03; 95% CI, 0.99–1.07).

**Table 2.** Immunization Rate by Professional Role, Location, Level of Patient Interaction and Facility

Variable	Baseline Immunization Rate, No. (%)	Intervention Season 1 Immunization Rate, No. (%)	Intervention Season 2 Immunization Rate, No. (%)	Incident Rate Ratio (95% CI)^a^	*P* Value
**Professional role**					
Nurses	1,891 (61.1)	2,258 (74.1)	2,224 (70.1)		
Allied health	385 (64.3)	460 (78.8)	427 (73)	1.10 (1.03–1.17)	.005
Other clinical^b^	1,179 (72.4)	1,312 (82)	1,310 (80)	1.10 (1.05–1.15)	<.0001
Support^b^	1,689 (48.3)	2,237 (68.6)	2,193 (68.6)	1.24 (1.03–1.49)	.02
**Location**					
Inpatient	3,267 (63.8)	3,775 (76.1)	3,723 (72.8)		
Ambulatory	615 (58)	815 (76)	797 (71)	1.02 (0.97–1.07)	.5
Administration	1,262 (47.7)	1,677 (68.1)	1,634 (69.4)	0.90 (0.84–0.97)	.004
**Patient interaction**					
Patient facing	3,478 (64.9)	4,059 (77)	3,989 (73.5)		
Non–patient facing	1,666 (48.1)	2,208 (68.5)	2,165 (68.4)	0.72 (0.60–0.87)	.0006
**Facility**					
Acute care	2,584 (56.2)	3,240 (72)	3,251 (69.9)		
Long-term care	648 (78.4)	670 (86.1)	593 (78.9)	1.24 (1.18–1.31)	<.0001
Rehabilitation	236 (55.7)	287 (71.8)	260 (63)	0.94 (0.87–1.02)	.1
Orthopedic hospital	189 (49.3)	245 (68.4)	259 (66.6)	0.93 (0.86–1.01)	.07
Undedicated	1,487 (57.3)	1,825 (74.2)	1791 (75)	1.20 (1.14–1.26)	<.0001
Overall^c^	5,144 (58.3)	6,267 (73.8)	6,154 (71.6)	1.24 (1.20–1.28)	<.0001

a First variable within category is the reference standard.

b Other clinical includes physicians, laboratory staff, medical imaging, observers, and recreation therapists. Support includes administrative staff, environmental services, plant operations/facility maintenance.

c After adjusting for facility, location, level of patient interaction and professional role.

## Discussion

A policy mandating an online learning module for HCWs intending to decline influenza immunization was associated with nearly a 25% relative increase in immunization and significant reduction in HAI prior to the COVID-19 pandemic.

There is a need for healthcare institutions to augment immunization rates beyond what may be achieved using promotional strategies alone.^5–7^ Mandating a decision by HCWs is feasible, but how best to implement the declination option remains unclear.^10^ Requiring an in-person interview to decline to be immunized may be associated with the greatest increase in HCW immunization, but this approach is resource intensive and not possible for larger institutions.^9^ Conversely, requiring that HCWs simply sign a declination statement fails to meaningfully nudge HCWs toward immunization.^7,9,10^ In either situation, resources may be diverted away from vaccine efforts in favor of the declination process itself if the process requires additional resources to implement or to track large numbers of declinations.^9^

By linking the declination statement to a mandatory online learning module, we ensured that all HCWs were making an informed choice, without infringing on HCW autonomy or requiring increased resources. The rise in HCW immunization was 20-fold greater than the number of employees who were vaccinated after completing the module, suggesting that it primarily functioned as an effective nudge toward the immunization option.

In the context of the current COVID-19 pandemic, expanding this strategy to the SARS-CoV-2 vaccine could address some of the vaccine hesitancy that has hampered early immunization efforts.^2^ A recent survey suggested that many HCWs are deferring their decision to be immunized due to uncertainties about the regulatory approval and protective capabilities of this vaccine.^4^ A similar approach to our influenza policy could bolster SARS-CoV-2 vaccination rates by nudging those who have deferred their decision.

Our study has important limitations. First, it is an uncontrolled before-and-after study that is subject to potential confounding. However, HCW immunization rates remained stable for several seasons prior to this intervention and increased immunization rates occurred across all subgroups. Second, adherence to the mandatory decision policy was not 100%, mainly due to the need to exempt HCWs who are on leave. Ongoing enforcement of this policy may be necessary to sustain the higher immunization rates beyond 2 years.

Our study outlines a novel approach to increasing HCW immunization through mandatory education for those intending to decline the influenza vaccine. In the absence of mandatory vaccination, this model of care may help to augment SARS-CoV-2 vaccine programs.
